# Determinants Associated With CD4 Cell Count and Disclosure Status Among First-Line Antiretroviral Therapy Patients Treated at Felege Hiwot Comprehensive Specialized Hospital, Ethiopia

**DOI:** 10.1155/jotm/5989447

**Published:** 2025-04-11

**Authors:** Abdela Assefa Bekele, Awoke Seyoum Tegegne, Nurye Seid Muhie

**Affiliations:** ^1^Department of Statistics, Assosa University, Assosa, Ethiopia; ^2^Department of Bio-Statistics, Bahir Dar University, Bahir Dar, Ethiopia; ^3^Department of Statistics, Mekdela Amba University, Tulu Awulia, Ethiopia

**Keywords:** adults, ART, CD4 cell count, determinants, disclosure status, first-line, HIV, retrospective

## Abstract

**Background:** In the last two decades, Human immune deficiency virus has been a major health concern in sub-Saharan Africa particularly in Ethiopia. The objective of this study was to identify determinants associated with CD4 cell count and disclosure status among first-line antiretroviral therapy patients treated at Felege Hiwot Comprehensive Specialized Hospital, Ethiopia.

**Methods:** This retrospective study was conducted at Felege Hiwot Comprehensive Specialized Hospital. Data analysis was conducted using Statistical System Analysis (SAS) software Version 9.4. In this study, quasi-Poisson mixed-effects model for CD4 cell count, a binary logistic regression model for disclosure status, and joint modeling were used.

**Result:** Out of 300 adult participants, around 76% of the patients were discloses their disease status to their family members. The correlation between CD4 cell count and disclosure was positive (0.4607). The current study indicates that among the predictor variables, noneducators (*β* = −0.6185, *p*-value < 0.01), primary educators (*β* = −0.3687, *p*-value < 0.01), employed patients (*β* = 0.3888, *p*-value < 0.01), adherent patients (*β* = 0.2274, *p*-value < 0.01), and patients who did not had social support (*β* = −0.1148, *p*-value = 0.030) have a significant effect for CD4 cell count. Similarly, noneducators (AOR = 0.000145, *p*-value < 0.01), primary educators (AOR = 0.004413, *p*-value < 0.01), employed patients (AOR = 3.4562, *p*-value = 0.021), adherent patients (AOR = 1.564, *p*-value < 0.01), and patients who did not had social support (AOR = 0.075, *p*-value = 0.0078) had a significant effect for disclosure status.

**Conclusion:** This study concluded that patients who had disclosed their disease status to near relatives or families have a positive correlation with CD4 cell count through time. This study also concluded that significant determinants affected both the variables of interest were educational level, occupation, adherence, and social support. Health professionals should give more attention to these important determinants to create good status of patients. In addition, health staff should conduct health-related studies for individuals to understand better ART follow-up. Patients should be adhere to their prescribed HIV medication properly on time and disclose their disease status without fearing stigma and discrimination to the community; this may help to increase their CD4 cell count. The family members should give social support to the infected patients, and the government should work on education; this may help to improve their CD4 cell count and increase the prevalence of disclosure of the disease status. The authors also recommended that further studies of this nature include other important variables that are not included in this study such as income of the patients and many other covariates.

## 1. Background

Globally, 79.3 million people are infected with HIV and 36.3 million people died from AIDS-related illnesses since the start of the epidemic. There were an estimated 37.7 million people living with HIV, and among these, 36 million people are adults and over two-thirds of them were in the sub-Saharan African region in 2020. Global trends in HIV infection show a general rise in the prevalence of the virus and significant association with AIDS-related mortality, which are mostly due to the benefits of antiretroviral therapy (ART) for survival [1].

Eastern and Southern Africa remain the HIV hotspots, with over 55% of HIV-positive people living. Despite the significant impact, the number of new infections in the region has fallen by 38% since 2010, yet the region continues to face a substantial challenge. Almost every country in the region is experiencing a broad HIV epidemic, with national HIV prevalence rates exceeding 1% [2].

In Ethiopia, patients bear about 70% of the global HIV infection burden. For more than 2 decades, HIV/AIDS has been a major health concern in sub-Saharan African countries. Ethiopia is one of those countries, with 620,000 HIV-/AIDS-positive people. With a prevalence rate of 1%, around 580,000 people are adults. About 23,000 people have been newly infected with HIV, and 11,000 have died as a result of AIDS-related sickness [2].

Then, the main issue pertaining to public health in Ethiopia is HIV infection; especially, the majority of infected individuals reside in the Amhara region. Between 2015 and 2018, the region recorded 57,293 new HIV cases, with the number of cases varying by time period, gender, age group, and location [3].

Immunological markers (CD4 cell count) are used in Ethiopia to decide on initiation of antiretroviral therapy and monitor HIV/AIDS disease progression [4]. CD4 cell count has been reported to have a strong association with progression to AIDS-related illness or death [5]. In addition, the measurement of CD4 cell counts is a strong predictor of progression to AIDS and a means of monitoring ART [6]. Disclosure of HIV status is considered to be a critical predictor of ART treatment [7]. Disclosure has become an important predictor to biological markers for CD4 cell count [3, 8, 9].

Disclosure of HIV status is used to improve immunological responses (CD4 cell count) to ART [10, 11] and increases the chance of viral suppression [12], and retention in care [13]. Then, disclosure of one's HIV status is a key aspect in HIV prevention and treatment programs [5]. It is the base for accessing care and treatment programs, gaining HIV-specific social support [6], attaining psychosocial support, reducing stigma, adhering to treatment, and promoting safer health [10, 11].

Therefore, disclosure changes the nature of the role of healthcare professionals [14] and is important to public health goal for a number of different reasons [15]. However, disclosure of HIV status may foster an environment that creates difficulties in patient adherence and retention in care [16]. Disclosure also carries important risks [17, 18] like the loss of privacy/confidentiality, the breakdown of relationships, loss of economic stability, and personal violence [19], social ostracism, physical harm, and workplace discrimination [20], and complex psychosocial challenges [21].

From different previous studies that were carried out separately, the determinant's significantly affected discloser statuses were types of ART, contraceptives' use [22], adherence to clinic visits [13], older age, patients living in an urban location, ART treatment adherence, viral load, mental health symptoms [23], monthly income, type of occupation, being divorced due to HIV status [10], sex, viral load suppression, having good ART adherence, perceiving stigma [11], higher educational, sexual partners, having follow-up counseling, being tested for HIV in antenatal care clinic [24], persons living with a significant other or friends, CD4 count < 200, receipt of social support [25], WHO Clinical Stage I, number of lifetime sexual partners [18], and functional support [26].

Similarly, determinants significantly affected CD4 cell count were baseline CD4 count, younger age, working functional status, time in treatment contributed [27], clinical stage, tuberculosis (TB) co-infection [28], hematocrit, platelet cell count, lymphocyte count, sex, adherence, [29], higher viral load [29], stavudine-based regimen, a low body weight, starting ART with a stavudine-based regimen [30], ownership of cell phone, marital status, residence area, level of disclosure of the disease to family members [31], baseline hemoglobin levels, and actively working patients [32].

As far as our understanding is concerned, no previous research has been conducted on identifying determinants associated with CD4 cell count and disclosure. Therefore, the objective of current investigation was to identify determinants associated with CD4 cell count and disclosure among first-line ART patients treated at Felege Hiwot Comprehensive Specialized Hospital (FHCSH), Ethiopia. The result obtained in current investigation helps for health-related education and to design interventional strategy.

## 2. Materials and Methods

### 2.1. Study Area and Population

The study was conducted at FHCSH. The study population for this study was adult HIV-positive patients under first-line ART regimen.

### 2.2. Study Design

Based on data from the ART clinic, a retrospective research design was used to recover relevant information from HIV/AIDS patients' medical records to achieve the potential risk factors affecting disclosure of HIV disease status and CD4 cell count progression.

### 2.3. Inclusion Criteria and Exclusion Criteria

The current investigation included all HIV-positive adults whose ages were 15 years and above and who started their treatment at FHCSH between January 1, 2016 and December 31, 2021 and had at least two follow-up times at this hospital. Patients under the age of 15 who are receiving ART therapy for refilling their prescriptions, as well as those who are not enrolled in an ART clinic in FHCSH or who were not enrolled throughout the study period would be excluded. In this investigation, patients with less than two follow-up periods were also excluded.

### 2.4. Sample Size Determination

The sample size was calculated using a single population proportion formula that accounts for the proportion of people who disclose their HIV disease status.

(1) $$\begin{array}{c} n_{0}=P^{*}{\left(1-P\right)}^{*}{\left(\frac{z_{\alpha /2}}{d}\right)}^{2}. \end{array}$$

If the value of *n*_0_/(*N* ) is less than 0.05, *n* is equal to *n*_0_ (*n* = *n*_0_), else *n* needs a satisfactory approximation to the sample size which is as follows:

(2) $$\begin{array}{c} n=\frac{n_{0}}{1+\left(n_{0}/N\right)}, \end{array}$$

where *N* is the target population and the total number of HIV/AIDS patients aged 15 and up on ART during the study period (*N*) = 1110. The sample fraction of disclosed patients in the population (*P*) = 0.473 from the previous study [3], and *α* is the level of significance which is 5%, and *z*_*α*/2_ is the value from the standard normal distribution indicating the confidence level that would be utilized, and *d* is the margin of error which is 5%. Finally, our sample size has been the computed simple size with a 5% of nonresponse rate is 300.

### 2.5. Data Extraction Procedures

The required data were extracted from each participants' chart using data extraction format. The format was developed by an author in consultation with health staffs.

### 2.6. Variables Under Investigation

#### 2.6.1. Response Variable

The longitudinal response variables for current study were CD4 cell count and disclosure status.

#### 2.6.2. Predictor Variables

In this study, the expected covariates that may influence CD4 cell count and disclosure status among adults living with HIV/AIDS under treatment were the following.


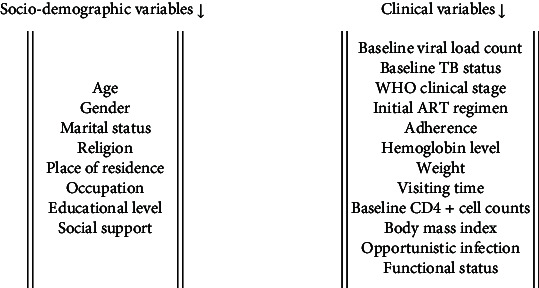


### 2.7. Exploratory Data Analysis (EDA)

EDA comprises generating summary statistics for numerical data in the dataset and creating various graphical representations in order to better understand the data. The individual profile plot, the mean profile plot, and variance structure would be considered as part of data exploration. To explore the individual profile, plot of the response with time was used to show whether there is a noticeable pattern common to most subjects. These individual profiles can also provide some information on within- and between-subject variability. The major purpose of exploring the mean structure is to choose the fixed effects for the model. To explore the overall mean, we plot the response variable against time including individual and overall mean profiles. In line with the overall mean, the possible differences between the groups will be studied by plotting the mean of each group separately with the same figure. Variance structure shows that the evolution of the variance is important to build an appropriate longitudinal model. Variance function is used to model nonsystematic variability.

### 2.8. Statistical Model

Three different models were used for the data analysis, namely a generalized linear mixed-effects model for the longitudinal data, a binary logistic regression model for the binary outcome data, and joint model of the two responses.

### 2.9. Method of Data Analysis

In this study, data analysis was conducted using Statistical System Analysis (SAS) software Version 9.4.

### 2.10. Method of Model and Covariance Structure Selection

The primary aim of model and covariance selection is to choose the simplest model and covariance structure that provides the best fit to the observed data. There are several methods of model and covariance structure selection. It can be easily selected by using the Akaike information criteria (AIC) and Bayesian information criteria (BIC).

## 3. Results

### 3.1. Sociodemographic Characteristics of Patients

Out of the total of 300 patients included in the study, 55% of the patients were female; from these, 77.57% of patients disclose their HIV status to their families. Out of the total samples included in this study, 59% of patients were unemployed, and 63.67% of patients were from urban areas. About 43.33% of patients have only completed their first year of school, and the majority of patients are married (36%). From a total sample, 57.33% got social support from the community, and 86.67% identified as practicing an orthodox religion. The remaining variable can be expressed as similar to the above (Table 1).

### 3.2. Clinical Characteristics of Patients

Out of 300 patients, 32% of patients do not adhere to their medication, and adherent patients have a higher number of CD4 cell mean (425.73) than nonadherent patients (225.33). When patients began ART, 23.3% were in Clinical Stage I, 37.7% were in Clinical Stage II, 20.3% were in Clinical Stage III, and the remaining were in Clinical Stage IV. Regarding BMI, 60.3% of the patients' body mass indices were normal, 24.7% were underweight, and 15% were overweight. A total of 47.7% of patients have opportunistic infections, and 25.3% of patients had TB infection. When we look at the patients' functional status, 72.3% of the patients were working functional status, 12.0% were bedridden, and 15.7% were ambulatory. The most common ART regimen at the (FHCSH) is TDF + 3TC + EFV ART regimen, which accounted 47.7% of the total sample. The remaining variable can be expressed as similar to the above (Table 2).

### 3.3. Count and Continuous Characteristics of Patients

The mean of baseline CD4 cell count and the standard deviation were 357.33 and 323.404, respectively, and the mean age and the standard deviation of the patients were 34.30 and 11.145, respectively. The patients' viral loads were a mean of 8959.60 and a standard deviation of 35,169.834 during the study period (Table 3).

Out of 300 total sample, 76.3% of the patients disclosed their HIV disease status to their families, whereas the remaining 23.7% of the patients are not disclosed their disease status, and the mean CD4 cell for all patients is 368.36 cells/mm^3^, whereas disclosed patients accounted for 493.54 cells/mm^3^ and nondisclosed patients accounted for 172.95 cells/mm^3^ (Table 4).

### 3.4. Exploring Individual Profile Plot

**Figure 1:**
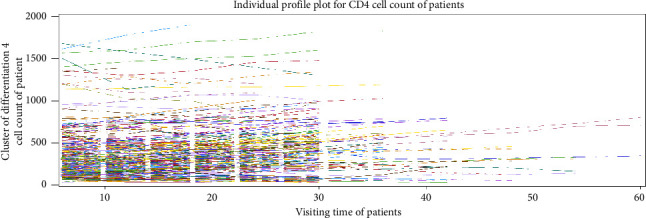
Individual profile plot of CD4 cell count of adult patients.

The individual profile plots of CD4 cell count change of HIV-infected patients over time is explored. It is used to observe the within- and between-subject variability across their CD4 cell count. From the plot, we observe that almost all patients have a slow increase in their CD4 cell count over time (Figure 1).

### 3.5. Exploring Mean Profile Plot

**Figure 2:**
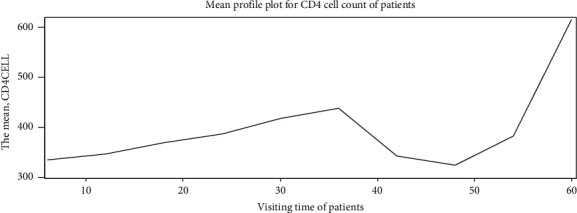
Mean profile plot for CD4 cell count with visiting time.

CD4 cells rose from the beginning (sixth) month up to the 36th month, started to decline up to the 42th month, and then began to climb fast up to the final month of the visit (60th) (Figure 2).

**Figure 3:**
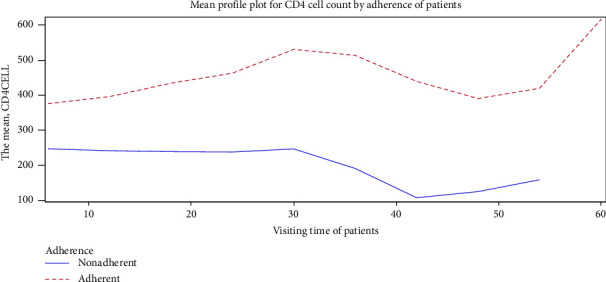
Mean profile plot for CD4 cell count by adherence.

Adherent patients experienced a considerably higher average CD4 cell count change over the follow-up times than nonadherent patients. The following figure shows the mean profile plot of CD4 cell count change by the adherence of patients over the follow-up durations (Figure 3).

**Figure 4:**
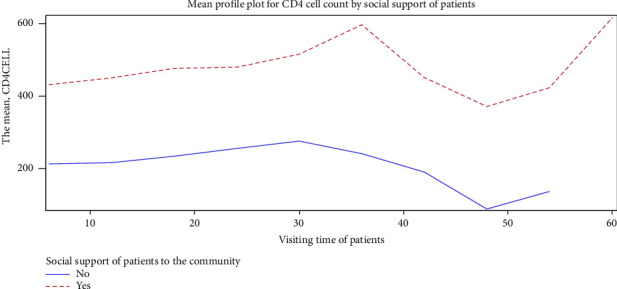
Mean profile plot for CD4 cell count by social support.

Socially supported patients experienced a much bigger average CD4 cell count change across the follow-up periods compared to unsupported patients (Figure 4).

### 3.6. Exploring Variance Function

**Figure 5:**
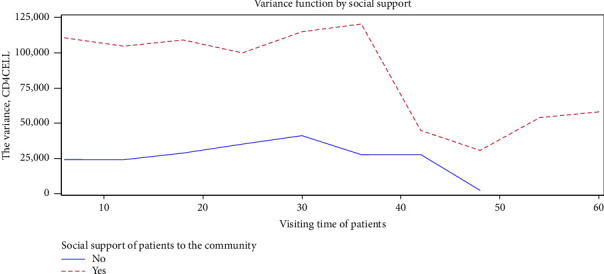
Variance function for CD4 cell count by social support.

High variation among socially supported patients is observed in Figure 5 at all times as compared with nonsupported patients. The variance of both groups increases at some point and decreases at another point, which suggests that there is no constant variance.

### 3.7. Variable Selection

In a univariate analysis of generalized linear mixed-effects model, gender, age, time, baseline CD4, weight, disclosure, educational level, occupation, adherence, WHO stage, social support, TB status, opportunistic infection, body mass index, baseline viral load, functional status, and the interaction effect of follow-up time, and educational level variables are significant at 25% level of significance for CD4 cell count. Similarly, in a univariate analysis of the binary logistic regression model, gender, age, residence, religion, marital status, educational level, occupation, social support, and functional status variables are significant at a 25% level of significance for disclosure. All significant variables in univariate analysis are included in a multivariate analysis.

### 3.8. Model Selection in a Generalized Linear Mixed Model

The three models in this study are the quasi-Poisson mixed-effects model, the Poisson mixed-effects model, and the negative binomial mixed-effects model. Our data's mean and variance are 364.93637 and 85,758 correspondingly, which shows that the data are too distributed. The Poisson mixed-effects model is not what we have. Consequently, the model is either a quasi-Poisson mixed-effects model or a negative binomial mixed-effects model. Using a smallest AIC, BIC, and −2 log-likelihood criterion, we have chosen the model that best fits our data. These quasi-Poisson mixed-effects models are the final models to fit our data, according to Table 5.

### 3.9. Selection of Covariance Structure in a Generalized Linear Mixed Model

Among the different covariance structures, unstructured had smallest AIC and BIC selected for data analysis (Table 6).

### 3.10. Selection of Random Effects in a Generalized Linear Mixed-effects Model

The random intercept and random slope models meet the AIC, BIC, and −2 log-likelihood criteria (Table 7).

### 3.11. Model Selection for Binary Response

As we know, a small AIC model would fit the data, which exhibited the model fit statistics for binary models and demonstrated that the intercept and covariates have small AICs (Table 8).

### 3.12. Joint Model Analysis for Binary and Count Data

Table 9 displays the parameter estimates, standard errors of estimates, and *p*-value for the longitudinal and binary submodels. From the longitudinal submodel, age, time, baseline CD4, educational level, occupation, adherence, WHO stage, social support, TB status, opportunistic infection, and body mass index were significantly related to CD4 cell count. From the binary submodel educational level, occupation, adherence, social support, opportunistic infection, and functional status were significantly related to the disclosure of disease status of HIV/AIDS patients.

Therefore, we can conclude that the determinants educational level, occupation, adherence, and social support jointly affected the change of CD4 cell count and disclosure of disease status. The correlation between CD4 cell count and disclosure of disease status was about 0.4607 which indicates a positive correlation between the two responses.

#### 3.12.1. Interpretations of Predictors Under Longitudinal Submodel

As patients' age increased by 1 year, the log of expected CD4 cell count was decreased by 0.0041 cells/mm^3^ (*p*-value = 0.0059), while all other variables remained constant. On the other hand, as visiting times of patients increased by one unit, the log of expected CD4 cells count was increased by 0.0072 cells/mm^3^ (*p*-value ≤ 0.0001). Correspondingly, in HIV-infected adults, the log of expected CD4 cell count was increased by 0.0379 cells/mm^3^ (*p*-value ≤ 0.0001) for a one unit increase in baseline CD4, keeping the other variables constant (Table 9).

The level of education also significantly affected the two variables of interest. Hence, the log of expected CD4 cell count for noneducated, primary, and secondary HIV-positive patients was decreased by 0.6185 cells/mm^3^ (*p*-value ≤ 0.01), 0.3687 cells/mm^3^ (*p*-value ≤ 0.0001), and 0.2693 cells/mm^3^ (*p*-value ≤ 0.0012), respectively, as compared to tertiary educational level of patients given that the other covariates were constant (Table 9).

The log of expected CD4 cell count for HIV-infected adults who were employed was significantly higher by 0.3888 cells/mm^3^ (*p*-value ≤ 0.0001) compared to that of unemployed patients, keeping the other variables constant. Patient's adherence status was also an another important variable for CD4 cell count, and the result showed that when comparing HIV-infected adults who had adhered to their prescribed medicine to those who had not, the log of expected CD4 cell count was significantly higher by 0.2274 cells/mm^3^ (*p*-value 0.0001), all other factors being held constant (Table 9).

The log of expected CD4 cell count for HIV-infected adults who had WHO Clinical Stage I, Stage II, and Stage III was significantly higher by 0.3709 cells/mm^3^ (*p*-value ≤ 0.0001),0.2661 cells/mm^3^ (*p*-value = 0.0007), and 0.1514 cells/mm^3^ (*p*-value = 0.0447), respectively, compared to patients who had WHO Clinical Stage IV, keeping the other variables constant. Also, the log of expected CD4 cell count for HIV-infected adults who did not get social support was significantly lower by 0.1148 cells/mm^3^ (*p*-value = 0.0303) compared to patients who got social support, keeping the other variables constant (Table 9).

The log of expected CD4 cell count for TB-infected HIV-positive adults was significantly decreased by 0.2991 cells/mm^3^ (*p*-value ≤ 0.0001) as compared to noninfected adult patients, keeping the other variables constant. The log of expected CD4 cell count for HIV-positive adults with nonopportunistic infections was significantly increased by 0.3062 cells/mm^3^ (*p*-value ≤ 0.0001) as compared to patients with opportunistic infectious disease, keeping the other variables constant. The log of expected CD4 cell count for normal and overweighted BMI HIV patients was increased by 0.1412 cells/mm^3^ (*p*-value = 0.0205) and 0.3441 cells/mm^3^ (*p*-value = 0.0041), respectively, as compared to underweighted HIV-positive patients, keeping the other variables constant (Table 9).

#### 3.12.2. Interpretations of Predictors Under Binary Submodel

Keeping all other determinants constant, the estimated odds of disclosed HIV adult patients was 0.000145; this indicates that no-education patients were 99.98% (*p*-value ≤ 0.0001) less likely to be disclosed as compared to those tertiary educational level of patients. Similarly, keeping all other predictors constant, the estimated odds of disclosed HIV adult patients was 0.0044; this indicates that primary patients were 99.56% (*p*-value = 0.0029) less likely to be disclosed as compared to those tertiary educational level of patients. Similarly, keeping all other predictors constant, the estimated odds of disclosed HIV adult patients was 138.698; this indicates that secondary patients were 138.698 (*p*-value = 0.0274) times more likely to be disclosed the disease status as compared to those tertiary educational level of patients (Table 9).

Keeping all other determinants constant, the estimated odds of disclosed HIV adult patients was 18.456; this indicates that employed patients were 18.456 (*p*-value = 0.0218) times more likely to be disclosed the disease status as compared to those unemployed patients. Likewise, keeping all other predictors constant, the estimated odds of disclosed HIV adult patients was 30.296; this indicates that medication-adherent HIV-positive adults were 30.296 (*p*-value = 0.0003) times more likely to be disclosed the disease status as compared to those nonadherent adult patients (Table 9).

Keeping all other determinants constant, the estimated odds of disclosed HIV adult patients were 0.076; this indicates that patients who did not get social support were 92.4% (*p*-value = 0.0078) less likely to be disclosed the disease status as compared to those who got social support patients. Similarly, keeping all other predictors constant, the estimated odds of disclosed HIV adult patients were 0.015; this indicates that ambulatory patients were 98.5% (*p*-value ≤ 0.0009) less likely to be disclosed as compared to those working patients. Similarly, keeping all other predictors constant, the estimated odds of disclosed HIV adult patients was 0.033; this indicates that bedridden patients were 96.7% (*p*-value = 0.0023) less likely to be disclosed the disease status as compared to those working patients (Table 9).

## 4. Discussion

In this study, 76% of adult patients had expressed the status of disease to family members or relatives. This indicated that majority of the patients had disclosed their disease status to family member. Then, HIV disclosure is a critical component to prevention and ART treatment efforts [6, 10, 16, 25]. This similarity might be due to the same study population.

The correlation between CD4 cell count and disclose status of the disease status is positive. This indicates that patient's immunity levels increase due to their discloser of the disease to family members or relatives. This means that patients expressed the disease to relatives increase proper follow-up ART and results for better CD4 cell count. The result of this study is in line with previous studies [7, 26, 28, 33]. However, this result is contradicted with the previous study in Uganda [8]. The potential reason for this variation might be the study area and study time, sample size and difference in methodology applied.

When the patients' visiting time increases, their corresponding CD4 cell count also increases and they encouraged disclosing the disease status. The potential reason for this might be the proper follow-up, which leads for being medication-adherent and this further leads to good health progressions like increase of CD4 cell count change. [4, 29, 31–34].This similarity might be due to the same predictors, and similar methodology.

Educated HIV-positive adults have higher number of CD4 cell count and they have more probability to disclose the disease status as compared to noneducated patients. This might occur because patients become more educated and they may have better care of their health and may have disclosed their disease status to family members, to take their medication properly on time and they may have enough understanding about ART, for this reason, the CD4 cell count [24, 35, 36]

Medication-adherent HIV-positive adults have higher number of CD4 cell count and such patients disclosed their disease status to family members more likely as compared to nonadherent HIV patients. This is the reason that series adherent patients show a good progress in reducing viral loads and increase of CD4 cell counts. This may be the case that using ART regularly improves adherence to healthcare services (with an emphasis on HIV status disclosure), which in turn raises awareness of HIV-positive status disclosure. Similarly, the odds of being disclosed the disease status of adherent patients is by far better as compared to nonadherent patients. Patients who disclosed their disease status can adhere to the medication on time irrespective of any other body living with them. [6, 7, 11, 23, 29, 36, 37]. This consistency might be due to the same predictors, similar study population and statistical methodology.

Occupation of HIV-positive adults also jointly affects the two response variables. The expected number of CD4 cell count for employed HIV-positive individuals is by far better as compared to unemployed ones. This result is consistent with other previously conducted researches [10, 24, 35]. The potential reason for this might be the fact that employed patients have better means of income for daily consumption of individuals. The disclosure of the disease status of employed HIV-positive individuals is better than nonemployed patients. The potential reason for this might be the fact that employed patients is forced to disclose the disease to get work leave or permission to be free from their regular work load. The idea of this study was similar with two former studies [38, 39].

This finding showed that participants who got social support from the community are better in CD4 cell count and such patients have high probability of disclosing their disease status to their families than those who had not get social support. The potential reason for this may be the fact that such patients might have good HIV medication (adherence), and they did not fear stigma and discrimination in disclosing the disease status. This result was also consistent with the result obtained from previous researches [7, 11, 16, 26, 40].

## 5. Conclusions and Recommendation

The result of this study concluded that patients who had disclosed their disease status to near relatives or families have a positive correlation with CD4 cell count through time. This study also concluded that significant determinants for both the variables of interest were educational level, occupation, adherence, and social support, jointly affected among HIV-positive adult patients.

Health professionals should give more attention to these important determinants to create good status of patients. In addition, health staff should conduct health-related studies for individuals to understand better ART follow-up. Patients should be adhere to their prescribed HIV medication properly on time, and disclose their disease status without fearing stigma and discrimination to the community; this may help to increase their CD4 cell count. The family members should give social support to the infected patients, and the government should work on education; this may help to improve their CD4 cell count and increase the prevalence of disclosure of the disease status. The authors also recommended that further studies of this nature include other important variables that are not included in this study like income of the patients and many other covariates.

## Figures and Tables

**Table 1:** Sociodemographic characteristics of adult patients.

Characteristics	Category	Disclosure status		Total (%)	CD4
		Disclosed	Not disclosed		Mean
Gender	Male	101	34	135 (45)	358.18
	Female	128	37	165 (55)	360.55

Residence	Rural	91	18	109 (36.33)	348.61
	Urban	138	53	191 (63.67)	365.64

Religion	Orthodox	194	66	260 (86.67)	357.97
	Muslin	18	2	20 (6.67)	307.59
	Other	17	3	20 (6.67)	439.42

Marital status	Never-married	78	16	94 (31.33)	372.58
	Married	80	28	108 (36.00)	355.29
	Widowed	29	11	40 (13.33)	349.03
	Divorced	42	16	58 (19.33)	365.36

Educational-level	No-education	25	34	59 (19.67)	228.52
	Primary	104	26	130 (43.33)	428.93
	Secondary	62	8	70 (23.33)	355.78
	Tertiary	38	3	41 (13.67)	367.31

Occupation	Unemployed	130	47	177 (59.00)	345.29
	Employed	99	24	123 (41.00)	377.64

Social support	No	61	67	128 (42.67)	243.88
	Yes	168	4	172 (57.33)	437.72

**Table 2:** Clinical characteristics of adult patients.

Characteristics	Category	Disclosure		Total (%)	CD4
		Disclosed	Not disclosed		Mean
Adherence	Nonadhere	65	31	96 (32.00)	225.33
	Adhere	164	40	204 (68.00)	425.73

WHO stage	Stage I	58	12	70 (23.33)	411.12
	Stage II	85	28	113 (37.67)	405.08
	Stage III	45	16	61 (20.33)	329.81
	Stage IV	41	15	56 (18.67)	236.37

ART regimen	AZT + 3TC + NVP	8	3	11 (3.66)	326.79
	AZT + 3TC + EFV	15	6	21 (7.00)	356.00
	TDF + 3TC + EFV	119	24	143 (47.67)	390.31
	TDF + 3TC + NVP	47	30	77 (25.67)	315.70
	TDF + 3TC + DTG	40	8	48 (16.00)	350.28

TB status	Uninfected	171	53	224 (74.67)	404.59
	Infected	58	18	76 (25.33)	235.63

Opportunistic infection	No	124	33	157 (52.33)	374.27
	Yes	105	38	143 (47.67)	343.48

Body mass index	Underweight	43	31	74 (24.67)	401.41
	Normal	164	17	181 (60.33)	348.51
	Overweight	22	23	45 (15.00)	360.49

Functional status	Ambulatory	11	36	47 (15.67)	364.22
	Bedridden	20	16	36 (12.00)	434.71
	Working	198	19	217 (72.33)	343.75

**Table 3:** Count and continuous characteristics for adult HIV-infected patients.

Mean age (std. deviation)	34.30 (11.145)
Mean baseline CD4 (std. deviation)	357.33 (323.404)
Mean weight (std. deviation)	54.57 (10.418)
Mean hemoglobin (std. deviation)	12.8945 (2.50608)
Mean viral load (std. deviation)	8959.60 (35, 169.834)

**Table 4:** Descriptive statistics for the diseases' disclosure status and CD4 cell count of patients.

Disclosure	Frequency	Percent	CD4 cell mean
Disclosed	229	76.3	493.54
Not disclosed	71	23.7	172.95
Total	300	100.0	368.36

**Table 5:** AIC, BIC, and −2 log-likelihood criteria for model selection.

Distribution	AIC	BIC	−2 log-likelihood
Negative binomial	16,676.57	16,817.32	16,600.57
Quasi-Poisson	15,893.82	16,027.16	15,821.82

**Table 6:** Comparison between different covariance structures in GLMMs.

Covariance structure	AIC	BIC
Independent	16,030.66	16,141.78
Compound symmetry	17,444.04	17,555.15
Unstructured	15,941.50	16,056.32
Autoregressive order 1	17,443.90	17,555.01
Toeplitz	17,444.33	17,555.45

**Table 7:** Selection of random-effect model to be included in the GLMM.

Random effect models	AIC	BIC	−2 log-likelihood
Random intercept	24,328.62	24,432.33	24,272.62
Random slope	33,386.40	33,490.11	33,330.40
Random intercept and random slope	15,939.01	16,050.12	15,879.01

**Table 8:** Model selection in a binary logistic regression model with disclosure response.

Criterion	Model fit statistics	
	Intercept only	Intercept and covariates
AIC	330.324	155.673
SC	334.028	214.933
−2 log L	328.324	123.673

**Table 9:** Parameter, estimate, and standard errors under the joint modeling analysis.

Parameters	CD4 cell count outcome			Disclosure status outcome		
	Estimate	Std error	*p*-value	Estimate	Std error	*p*-value
Intercept	5.0658	0.1526	< 0.0001	−1.5491	3.1354	0.6214
Age	−0.00417	0.002122	0.0059⁣^∗^	0.1336	0.04852	0.0597
Time	0.007219	0.000674	< 0.0001⁣^∗^			
Baseline CD4	0.0379	0.00542	< 0.0001⁣^∗^	0.001274	0.001583	0.4211
Educational level (ref = Tertiary)						
No-education	−0.6185	0.1097	< 0.0001⁣^∗^	−8.8371	1.1200	< 0.0001⁣^∗^
Primary	−0.3687	0.09477	0.0001⁣^∗^	−5.4231	0.8201	0.0029⁣^∗^
Secondary	−0.2693	0.08320	0.0012⁣^∗^	4.9323	0.2350	0.0274⁣^∗^
Occupation (ref = unemployed)						
Employed	0.3888	0.07032	< 0.0001⁣^∗^	2.9154	0.2701	0.0218⁣^∗^
Adherence (ref = nonadhere)						
Adhere	0.2274	0.05203	< 0.0001⁣^∗^	3.4110	0.9334	0.0003⁣^∗^
WHO stage (ref-stage IV)						
Stage I	0.3709	0.08991	< 0.0001⁣^∗^	1.5327	1.7376	0.3778
Stage II	0.2661	0.07863	0.0007⁣^∗^	−0.5980	1.4230	0.6743
Stage III	0.1514	0.07539	0.0447⁣^∗^	−0.01559	1.3192	0.9906
Social support (ref = yes)						
No	−0.1148	0.05295	0.0303⁣^∗^	−2.5836	0.6707	0.0078⁣^∗^
TB status (ref = uninfected)						
Infected	−0.2991	0.07015	< 0.0001⁣^∗^	0.4544	2.1284	0.8310
Opportunistic infection (ref = yes)						
No	0.3062	0.07242	< 0.0001⁣^∗^	0.5000	1.4173	0.7243
BMI (ref = underweight)						
Normal	0.1412	0.06088	0.0205⁣^∗^	−2.3619	1.2089	0.0509
Overweight	0.3441	0.1196	0.0041⁣^∗^	−0.8102	2.2669	0.7208
Functional status (working)						
Ambulatory	−0.01528	0.06393	0.8112	−4.2323	0.2780	0.0009⁣^∗^
Bedridden	−0.00987	0.06255	0.8746	−3.3997	0.1128	0.0023⁣^∗^

**Variance component**	**Estimate**			**Std error**	**95% CI**	

Var. R.I (CD4 cell)	0.1270			0.01099	(0.1005, 0.14)	
Var. R.I (disclosure)	12.9709			0.5508	(9.6734, 17.85)	
Corr. between the R.I	0.4607					

Abbreviations: Corr, correlation; ref, reference; R.I, random intercept; Std error, standard deviation.

⁣^∗^Variables that are significant at 5% of significance level.

## Data Availability

The data that support the findings of this study are available from the corresponding author upon reasonable request.

## Nomenclature

AIC: Akaike information criteria
AIDS: Acquired immune deficiency syndrome
ART: Antiretroviral therapy
BIC: Bayesian information criteria
CD4: Cluster differentiation 4
EDA: Exploratory data analysis
FHCSH: Felege Hiwot Comprehensive Specialized Hospital
GLMM: Generalized linear mixed model
HIV: Human immune deficiency virus
SAS: Statistical System Analysis
WHO: World Health Organization

## Supporting Information

The estimated subject-specific variability was statistically significant. The amount of variability among patients due to the effect of visiting time was 0.000467, and the correlation was −0.6241; this indicates that there is a negative correlation between the intercept and slope (visiting time). The estimates of the parameters in the separate and joint models are approximately similar to each other but not identical except that a change was observed in some parameters of predictors.

## References

[1] UNAIDS (2021). Fact Sheet 2021 Global HIV Statistics. *Ending the AIDS Epidemic*.

[2] UNAIDS and AIDSinfo (2021). Country Factsheets Democratic Republic of Congo 2020 HIV and AIDS Estimates Adults and Children Living With Country Factsheets DRC: 2020 HIV Testing and Treatment Cascade People Living With HIV Coverage of Adults and Children. *Unaids*.

[3] Tegegne A. S. (2023). Predictors of Adherence and Disclosure of HIV Status to Sexual Partners Among HIV Positive Adults Under HAART in Amhara. *North-West Ethiopia; Application of Joint Models*.

[4] MacArthur R. D., Perez G., Walmsley S., Baxter J. D., Mullin C. M., Neaton J. D. (2005). Comparison of Prognostic Importance of Latest CD4+ Cell Count and HIV RNA Levels in Patients With Advanced HIV Infection on Highly Active Antiretroviral Therapy. *HIV Clinical Trials*.

[5] The Opportunistic Infections Project Team of the Collaboration of Observational HIV Epidemiological Research in Europe Cohere in EuroCoord (2012). CD4 Cell Count and the Risk of AIDS or Death in HIV-Infected Adults on Combination Antiretroviral Therapy With a Suppressed Viral Load: A Longitudinal Cohort Study From COHERE. *PLoS Medicine*.

[6] Miranda-Filho B., Ce C. C., Aguiar F., Arraes R., Ximenes D. A. (2014). Factors Related to Changes in CD4 + T-Cell Counts Over Time in Patients Living With HIV/AIDS. *A Multilevel Analysis*.

[7] Mi T., Li X., Zhou G., Qiao S., Shen Z., Zhou Y. (2020). HIV Disclosure to Family Members and Medication Adherence: Role of Social Support and Self-Efficacy. *AIDS and Behavior*.

[8] Mwesigire D. M., Martin F., Seeley J., Katamba A. (2015). Relationship Between CD4 Count and Quality of Life over Time Among HIV Patients in Uganda: A Cohort Study. *Health and Quality of Life Outcomes*.

[9] Obermeyer C. M., Baijal P., Pegurri E. (2011). Facilitating HIV Disclosure Across Diverse Settings: A Review. *American Journal of Public Health*.

[10] Alhassan R. K., Nutor J. J., Gyamerah A. (2023). Predictors of HIV Status Disclosure Among People Living With HIV (PLHIV) in Ghana: The Disclosure Conundrum and Its Policy Implications in Resource Limited Settings. *AIDS Research and Therapy*.

[11] Melis Berhe T., Lemma L., Al A., Ajema D., Glagn M., Dessu S. (2020). HIV-Positive Status Disclosure and Associated Factors Among HIV-Positive Adult Patients Attending Art Clinics at Public Health Facilities of Butajira Town, Southern Ethiopia. *AIDS Research and Treatment*.

[12] Elizabeth Glaser Pediatric Aids foundation (2018). *Disclosure of HIV Status Toolkit for Pediatric and Adolescent Populations*.

[13] Izudi J., Okoboi S., Lwevola P., Kadengye D., Bajunirwe F. (2021). Effect of Disclosure of HIV Status on Patient Representation and Adherence to Clinic Visits in Eastern Uganda: A Propensity-Score Matched Analysis. *PLoS One*.

[14] Ryan S., Phillips M. (2021). HIV Disclosure-Professional Body Guidelines, the Law and the Boundaries of Medical Advice. *Medical Law Review*.

[15] Who (2024). HIV Status Disclosure to Sexual Partners: WHO Document Summa and Outcomes. *World Health Organization*.

[16] Lepira B., Mutombo P. B., Tylleskar T., Ali M. M. (2017). Disclosure of HIV Status and Its Impact on the Loss in the Follow-Up of HIV-Infected Patients on Potent Anti-Retroviral Therapy Programs in a (Post-) Conflict Setting: A Retrospective Cohort Study From Goma. *Democratic Republic of Congo*.

[17] Thapa S., Hannes K., Buve A., Bhattarai S., Mathei C. (2018). Theorizing the Complexity of HIV Disclosure in Vulnerable Populations: A Grounded Theory Study. *BMC Public Health*.

[18] Tessema B. T., Bune G. T., Mamo Z. B. (2023). Non-Disclosure of HIV-Positive Serostatus: Unmatched Case–Control Study in People Living With HIV in Public Health Facilities of Gedeo Zone, Southern Ethiopia. *HIV*.

[19] Derlega V., Winstead B., Folk-Barron L. (2014). Reasons for and Against Disclosing HIV-Seropositive Test Results to an Intimate Partner: A Functional.

[20] Chaudoir S. R., Fisher J. D., Simoni J. M. (2011). Understanding HIV Disclosure: A Review and Application of the Disclosure Processes Model. *Social Science and Medicine*.

[21] Jia W., Jiao K., Ma J. (2022). HIV Infection Disclosure, Treatment Self-Efficacy and Quality of Life in HIV-Infected MSM Receiving Antiretroviral Therapy. *BMC Infectious Diseases*.

[22] Damian D. J., Ngahatilwa D., Fadhili H. (2019). Factors Associated With HIV Status Disclosure to Partners and Its Outcomes Among HIV-Positive Women Attending Care and Treatment Clinics at Kilimanjaro Region, Tanzania. *PLoS One*.

[23] Edun O., Shenderovich Y., Zhou S. (2022). Predictors and Consequences of HIV Status Disclosure to Adolescents Living With HIV in Eastern Cape, South Africa: A Prospective Cohort Study. *Journal of the International AIDS Society*.

[24] Reis R. K., Sousa L. R. M., Melo E. S., Fernandes N. M., Sorensen W., Gir E. (2021). Predictors of HIV Status Disclosure to Sexual Partners Among People Living With HIV in Brazil. *AIDS and Behavior*.

[25] Elopre L., Westfall A. O., Mugavero M. J. (2016). Predictors of HIV Disclosure in Infected Persons Presenting to Establish Care. *AIDS and Behavior*.

[26] Jorjoran Shushtari Z., Sajjadi H., Forouzan A. S., Salimi Y., Dejman M. (2014). Disclosure of HIV Status and Social Support Among People Living With HIV. *Iranian Red Crescent Medical Journal*.

[27] Gezie L. D. (2016). Predictors of CD4 Count Over Time Among HIV Patients Initiated ART in Felege Hiwot Referral Hospital, Northwest Ethiopia: Multilevel Analysis. *BMC Research Notes*.

[28] Farhadian M., Mohammadi Y., Mirzaei M., Shirmohammadi-Khorram N. (2021). Factors Related to Baseline CD4 Cell Counts in HIV/AIDS Patients: Comparison of Poisson, Generalized Poisson and Negative Binomial Regression Models. *BMC Research Notes*.

[29] Muhie N. S., Tegegne A. S. (2023). Predictors for CD4 Cell Count and Hemoglobin Level With Survival Time to Default for HIV Positive Adults Under ART Treatment at University of Gondar Comprehensive and Specialized Hospital, Ethiopia. *BMC Research Notes*.

[30] de Castilla D. L. (2007). Predictors of CD4+ Cell Count Response and of Adverse Outcome Among HIV-Infected Patients Receiving Highly Active Antiretroviral Therapy in a Public Hospital in Peru.

[31] Seyoum A., Ndlovu P., Temesgen Z. (2017). Joint Longitudinal Data Analysis in Detecting Determinants of CD4 Cell Count Change and Adherence to Highly Active Antiretroviral Therapy at Felege Hiwot Teaching and Specialized Hospital, North-West Ethiopia (Amhara Region). *AIDS Research and Therapy*.

[32] Reda A., Biadgilign S., Deribew A., Gebre B., Deribe K. (2013). Predictors of Change in CD4 Lymphocyte Count and Weight Among HIV Infected Patients on Anti-Retroviral Treatment in Ethiopia: A Retrospective Longitudinal Study. *PLoS One*.

[33] Gebrerufael G. G. (2023). Predictors Associated With CD4 Cell Count Changes Over Time Among HIV-Infected Children on Anti-Retroviral Therapy Follow-Up in Mekelle General Hospital, Northern Ethiopia, 2019: A Retrospective Longitudinal Study. *BMC Pediatrics*.

[34] Stirrup O. T., Copas A., Phillips A. (2018). Predictors of CD4 Cell Recovery Following Initiation of Antiretroviral Therapy Among HIV-1 Positive Patients With Well-Estimated Dates of Seroconversion. *HIV Medicine*.

[35] Bayabil S., Seyoum A. (2021). Joint Modeling in Detecting Predictors of CD4 Cell Count and Status of Tuberculosis Among People Living With HIV/AIDS Under HAART at Felege Hiwot Teaching and Specialized Hospital, North-West Ethiopia. *HIV*.

[36] Nura G. J., Guyo A. B., Erango M. A. (2024). Determinants of Longitudinal Changes of CD4 Cell Count and Survival Time to Death of HIV/AIDS Patients Treated at Yabelo General Hospital, the Case of Pastoralist Area: Using Joint Modelling Approach. *PLoS One*.

[37] Tegegne A. S., Muluneh M. W., Agegn S. B., Biresaw H. B. (2022). A Comparison of Adherence and CD4 Cell Count With Respect to Virologic Failure Among HIV-Infected Adults Under Combination Antiretroviral Therapy (cART) at Felege Hiwot Teaching and Specialized Hospital, Bahir Dar, Ethiopia. *HIV*.

[38] Muhie N. S. (2024). Common Risk Factors for CD4 Cell Count and Hemoglobin Level Among Female Adult HIV-Positive Patients: A Retrospective Longitudinal Study. *Journal of Tropical Medicine*.

[39] Muhie N. S., Tegegne A. S. (2024). Determinants of Hemoglobin Level and Time to Default From Highly Active Antiretroviral Therapy (HAART) for Adult Clients Living With HIV Under Treatment: A Retrospective Cohort Study Design. *Scientific Reports*.

[40] Eustace R. W., Ilagan P. R. (2010). HIV Disclosure Among HIV Positive Individuals: A Concept Analysis. *Journal of Advanced Nursing*.

