# Supplementary material for: Determinants Associated With CD4 Cell Count and Disclosure Status Among First-Line Antiretroviral Therapy Patients Treated at Felege Hiwot Comprehensive Specialized Hospital, Ethiopia
Source: J Trop Med. 2025 Apr 11;2025:5989447. doi: 10.1155/jotm/5989447 (PMC12009679; doi:10.1155/jotm/5989447)
Supplement: Supporting Information — Additional supporting information can be found online in the Supporting Information section. [file 5989447.f1.docx]

#### Multivariate Analysis for Generalized Linear Mixed Effect Model

A multivariate analysis of the generalized linear mixed effect model (Table 1) displayed that age, time, baseline CD4 cell, weight, disclosure, educational level, occupation, adherence, WHO stage, social support, TB status, opportunistic infection, baseline viral load, functional status, and the interaction effect of follow up time and educational level variables were significantly associated with the log of expected CD4 cell count of HIV infected adults at 5% level of significance. The estimated subject-specific variability was statistically significant. The amount of variability among patients due to the effect of visiting time was 0.000467 and the correlation was -0.6241, this indicates that there is a negative correlation between the intercept and slope (visiting time).

Table 1. Estimate, Standard error, and 95% CI under the GLMM analysis including disclosure as a linear predictor

| **Effect** | **category** | **Estimates** | | **Std errors** | **95% CI** | | | **P-Value** |
| --- | --- | --- | --- | --- | --- | --- | --- | --- |
|  |  |  |  |  | **Lower** | **Upper** | |  |
| Intercept |  | 4.8756 | | 0.2106 | 4.4611 | 5.2901 | | <.0001 |
| Gender (ref=male) | Female | -0.01569 | | 0.05324 | -0.1202 | 0.08882 | | 0.7682 |
| Age |  | -0.00744 | | 0.002359 | -0.01207 | -0.00281 | | 0.0017* |
| Time |  | 0.005296 | | 0.001347 | 0.002646 | 0.007946 | | 0.0001* |
| Baseline CD4 |  | 0.000526 | | 0.000092 | 0.000346 | 0.000707 | | <.0001* |
| Weight |  | 0.00470 | | 0.002080 | 0.000621 | 0.008788 | | 0.0240* |
| Baseline viral load |  | -0.001345 | | 0.000012 | -0.0279 | -0.00603 | | <.0001* |
| Disclosure(ref=not disclosed) | Disclosed | 0.5084 | | 0.06781 | 0.3753 | 0.6415 | | <.0001* |
| Educational level (ref=Tertiary) | No-education  Primary  Secondary | -0.3720 | | 0.1115 | -0.5909 | -0.1530 | | 0.0009* |
|  |  | -0.2567 | | 0.09359 | -0.4404 | -0.07301 | | 0.0062* |
|  |  | -0.4044 | | 0.09764 | -0.5960 | -0.2127 | | <.0001* |
| Occupation(ref=unemployed) | Employed | 0.2062 | | 0.04958 | 0.003036 | 0.4089 | | <.0001* |
| Adherence(ref= non-adhere) | Adhere | 0.1339 | | 0.06400 | 0.008248 | 0.2595 | | 0.0368* |
| WHO stage(ref- stage IV) | Stage I  Stage II  Stage III | 0.5712 | | 0.09709 | 0.3806 | 0.7618 | | <.0001* |
|  |  | 0.4268 | | 0.08560 | 0.2588 | 0.5949 | | <.0001* |
|  |  | 0.2478 | | 0.08388 | 0.08310 | 0.4124 | | 0.0032* |
| BMI(ref=under weight) | Normal  Over weight | 0.07015  0.1709 | | 0.07390  0.1539 | -0.07493  -0.1312 | 0.2152  0.4729 | | 0.3428  0.2672 |
| TB status(ref=uninfected) | Infected | -0.2035 | | 0.06066 | -0.3226 | -0.08443 | | 0.0008* |
| Opp infection (ref=yes) | No | 0.3001 | | 0.05507 | 0.1920 | 0.4082 | | <.0001* |
| Functional status(ref=working) | Ambulatory  Bedridden | 0.07860 | | 0.08187 | -0.08212 | 0.2393 | | 0.3373 |
|  |  | 0.1807 | | 0.07225 | 0.03884 | 0.3225 | | 0.0126* |
| Social support (ref=yes) | No | -0.1981 | | 0.05567 | -0.3073 | -0.08878 | | 0.0004* |
| V-Time*Educational level (ref=Tertiary) | V-Time*0  V-Time*1  V-Time*2 | -0.00012  0.005965  0.01208 | | 0.004475  0.004101  0.004185 | -0.00890  -0.00209  0.003865 | 0.008670  0.01402  0.02030 | | 0.9795  0.1462  0.0040* |
| **Random effect** |  | **Estimates** | **Std errors** | | | | **P-value** | |
| Intercept($b_{oi}$)  Time($b_{1i}$)  Cor${(b}_{oi},b_{1i}$) |  | 0.2687  0.000467  -0.6241 | 0.02547  0.000043 | | | | <.0001  <.0001 | |

**Note:** $b_{oi} and$ $b_{1i}$ are the intercept and slope of the random effect of the model respectively and *indicates that variables that are significant at 5% of significance level

**Key:** cor is correlation of the random intercept, V-Time is visiting time, std error is standard error, 0 = no-education, 1= primary, 2 = secondary, Opp is opportunistic infection

#### Multivariate Analysis for binary logistic regression model

Table 2 indicates that residence, marital status, educational level, occupation, social support and functional status variables in the model are significantly improve the model fit.

Table 2. Binary logistic regression estimate, Standard error, adjusted odds ratio and 95% CI

| **Parameter** | **Estimate(β)** | **Std Error** | **Wald Chi-Square** | **AOR (95% CI)** | **P - value** |
| --- | --- | --- | --- | --- | --- |
| Intercept | 7.9518 | 2.3303 | 11.6437 | 2840.68 | 0.0006 |
| Gender (ref= Male)  Female | 0.4089 | 0.5060 | 0.6530 | 1.505(0.558, 4.058) | 0.4190 |
| Age | -0.00764 | 0.0252 | 0.0923 | 0.992(0.945, 1.043) | 0.7613 |
| Residence (ref= urban)  Rural | 1.6283 | 0.5891 | 7.6402 | 5.095(1.606, 16.166) | 0.0057* |
| Religion (ref= other)  Muslim  Orthodox | 0.1374  -0.6328 | 1.7229  1.3128 | 0.0064  0.2324 | 1.147(0.039, 33.586)  0.531(0.041, 6.961) | 0.9364  0.6298 |
| Functional Status (ref= working)  Ambulatory  Bedridden | -3.1760  -2.5810 | 0.6291  0.6625 | 25.4842  15.1787 | 0.042(0.012, 0.143)  0.076(0.021, 0.277) | <.0001*  <.0001* |
| Marital Status (ref= widowed)  Divorced  Married  Never-married | -0.0197  -0.5365  2.1233 | 0.7997  0.8232  0.8597 | 0.0006  0.4247  6.0999 | 0.981(0.205, 4.700)  0.585(0.116, 2.936)  8.358(1.550, 45.070) | 0.9804  0.5146  0.0135* |
| Educational Level (ref= tertiary)  No-education  Primary  Secondary | -4.7875  -2.1073  -1.2588 | 1.4062  1.1223  1.2050 | 11.5901  3.5254  1.0913 | 0.008(0.001, 0.131)  0.122(0.013, 1.097)  0.284(0.027, 3.013) | 0.0007*  0.0604  0.2962 |
| Occupation (ref= Unemployed)  Employed | 1.3311 | 0.6687 | 3.9628 | 3.785(1.071, 13.80) | 0.0465* |
| Social Support (ref=Yes)  No | -4.0866 | 0.7587 | 29.0080 | 0.017(0.004, 0.074) | <.0001* |

### **Note:***indicates that variables that are significant at 5% of significance level

**Key:** ref is reference, AOR is adjusted odds ratio, Std Error is standard error

The estimates of the parameters in the separate and joint models are approximately similar to each other but not identical except a change was observed in some parameters of predictors.
